# Zebrafish: a key model for unraveling endocrine-disrupting effects on thyroid development and function

**DOI:** 10.3389/fphar.2026.1746017

**Published:** 2026-02-18

**Authors:** Nathana Mezzalira, Vinicius Gonçalves Rodrigues, Caroline Serrano-Nascimento

**Affiliations:** 1 Laboratório de Fisiologia e Fisiopatologia da Tireoide (LaFFT), Departamento de Ciências Biológicas, ICAQF, UNIFESP, Diadema, Brazil; 2 Laboratório de Endocrinologia Molecular e Translacional (LEMT), Departamento de Medicina, Escola Paulista de Medicina, UNIFESP, São Paulo, Brazil

**Keywords:** endocrine disruptors, HPT axis, thyroid, thyroid hormones, zebrafish

## Abstract

The zebrafish (*Danio rerio*) has emerged as a crucial vertebrate model for studying thyroid physiology and toxicology, owing to its high genomic homology with mammals, external embryogenesis, transparent development, and the availability of advanced genetic manipulation techniques. Moreover, this experimental system is widely used for toxicity testing of environmental chemical compounds that affect thyroid function, yielding well-characterized phenotypic and molecular responses. It is well known that thyroid hormones regulate embryonic development in vertebrates, particularly the development of the central nervous system. Moreover, thyroid hormones control energy metabolism, growth, cellular differentiation, and overall homeostasis, physiological processes conserved in both zebrafish and mammal models. Therefore, this mini-review provides a comprehensive analysis of the current literature regarding the use of zebrafish to investigate the effects of endocrine-disrupting chemicals on the hypothalamic-pituitary-thyroid axis and thyroid function, as well as the associated physiological and behavioral responses. This review also discusses the limitations of the zebrafish model, including the challenges of establishing exposure models that are realistic and comparable to those experienced by humans and other animals in the environment. Finally, it discusses the intra- and intergroup variability, technical challenges in molecular analyses at early developmental stages, anatomical differences relative to humans, and difficulties in experimental handling and reproducibility. Nevertheless, zebrafish is an undeniable, versatile, and powerful model for advancing research in thyroid endocrinology and toxicology, especially during critical developmental windows, which are more complex to assess in mammals.

## 1 Introduction

### 1.1 Zebrafish as a model to assess thyroid function

**FIGURE 1:**
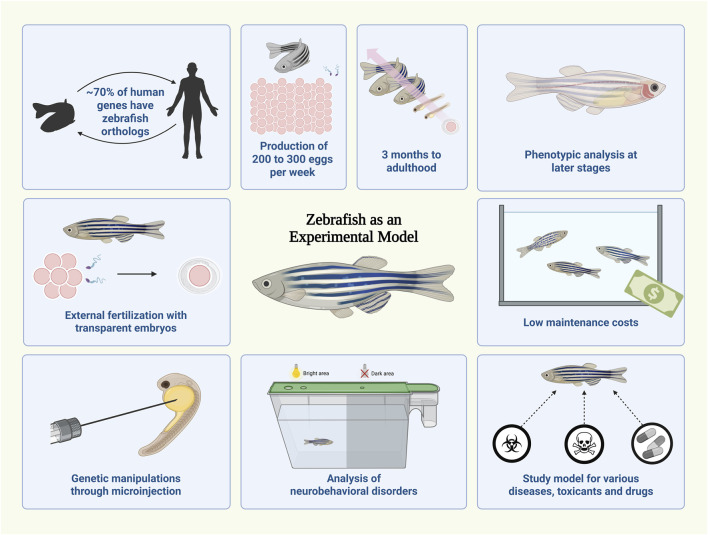
Advantages of using zebrafish as an experimental model. The zebrafish (*Danio rerio*) exhibits a high degree of genetic similarity to humans, supporting its broad applicability in biomedical research. Zebrafish reproduce prolifically (200–300 eggs per week) and reach adulthood in about 3 months. External fertilization and embryo transparency enable direct phenotypic observation and experimental manipulation. Genetic modifications can be efficiently performed through microinjection techniques, allowing functional studies from the earliest developmental stages. Zebrafish is also widely used for behavioral and neurobiological protocols, serving as a versatile model for studying various diseases, toxic compounds, and drugs, while maintaining low maintenance and husbandry costs in comparison to rodent models. Created in BioRender.

The zebrafish (*Danio rerio*) is a small, highly fecund tropical freshwater fish whose rapid development and embryonic transparency enable efficient phenotypic screening and embryological studies. Its high amenability to genetic manipulation, together with strong genomic conservation with humans (≈70% orthology), confers high translational relevance (Choi et al., 2021). These features, combined with low maintenance costs, ease of handling, and reduced ethical constraints during early development, establish zebrafish as a robust alternative to rodent models for studying endocrine system dysregulation (Figure 1).

**FIGURE 2:**
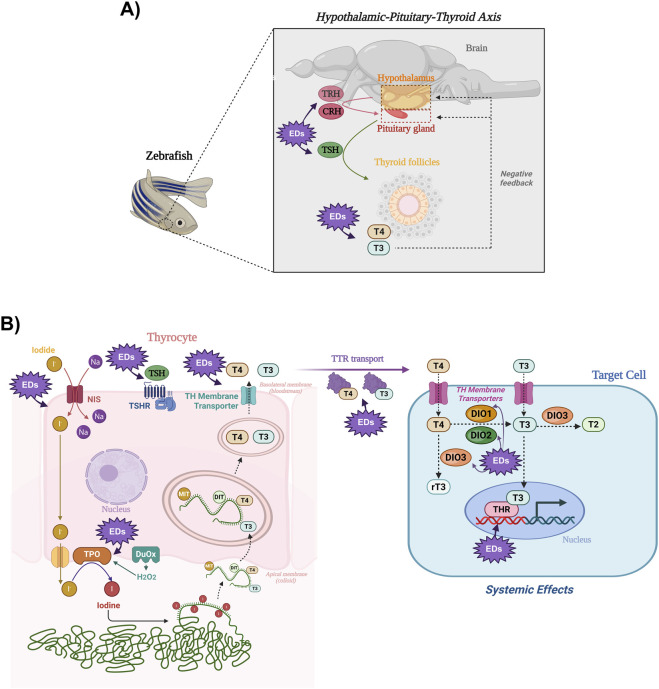
Schematic representation of the HPT axis, TH synthesis, transport, and action in zebrafish. **(A)** Hypothalamic TRH and CRH stimulate the pituitary to secrete TSH, which acts on thyroid follicles, stimulating the synthesis and release of T_3_ and T_4_, which regulate hypothalamus and pituitary function through negative feedback loops. **(B)** In the thyrocyte, TSH binds to its receptor (TSHR) and stimulates all the steps involved in the synthesis and secretion of TH. The sodium/iodide symporter (NIS) mediates iodide uptake, driven by the Na^+^ gradient generated by the Na^+^/K^+^-ATPase. Iodide is then transported to the follicular lumen, where the apical machinery, including thyroid peroxidase (TPO), catalyzes the oxidation of iodide to iodine and its incorporation into thyroglobulin (TG). Subsequently, TPO catalyzes the coupling reactions of iodotyrosines to generate T_4_ and T_3_. Iodinated TG is endocytosed and hydrolyzed in the cytoplasm, releasing TH, which exit the cell through basolateral membrane transporters and circulate bound to carrier proteins (e.g., transthyretin, TTR). In target tissues, TH are transported across the membrane through specific transporters, and deiodinases such as DIO2 and DIO1 convert T_4_ into the active T_3_, whereas DIO3 catalyzes the inactivation of T_3_ to T_2_ or T_4_ to rT_3_. T_3_ binds to nuclear TH receptors (THR), modulating gene transcription and eliciting systemic physiological effects. The purple starburst callouts highlight steps within the HPT axis and in TH synthesis, secretion, transport, metabolism, and action that have been reported as targets of endocrine-disrupting chemicals. Created in BioRender.

Zebrafish exhibit a highly conserved hypothalamic-pituitary-thyroid (HPT) axis; however, unlike mammals, in which TRH drives TSH release, TSH regulation in many teleosts appears to rely mainly on CRH, with TRH playing a limited, and possibly species-dependent role (Deal and Volkoff, 2020). Conversely, TH synthesis in zebrafish closely resembles that of mammals, including TSH-regulated hormone secretion. In circulation, TH are partly transported by functionally conserved transthyretin (TTR). At the tissue level, local TH availability is mainly controlled by deiodinases, with T3 acting through nuclear TH receptors across multiple zebrafish tissues to regulate cardiac, nervous, and reproductive systems (Figure 2).

Another highly conserved step between zebrafish and mammals is thyroid organogenesis, which begins around 20 h post-fertilization (hpf) and proceeds through the sequential expression of key transcription factors that drive thyroid specification, placode formation, and differentiation (Opitz et al., 2013; Marelli et al., 2021). Functional follicular cells appear around 55 hpf, followed by thyroid tissue proliferation and migration as dispersed follicles along the ventral aorta. TH production is detectable by 70–80 hpf and peaks during early larval development (Yadav et al., 2022).

Finally, the combination of embryonic transparency with gene editing, live imaging, and single-cell RNA-seq enables real-time analysis of thyroid morphogenesis and identification of key regulatory genes in zebrafish, a powerful and largely unfeasible approach in mammals for studying environmental disruption during critical developmental windows (Trubiroha et al., 2018; Gillotay et al., 2020). Collectively, this evidence establishes zebrafish as a powerful and versatile model for studying thyroid physiology, development, and environmental toxicology in vertebrates.

### 1.2 Zebrafish and thyroid disruptors

Endocrine disruptors (EDs) are largely man-made chemicals that interfere with endocrine function and are often persistent, lipophilic, and bioaccumulative. Alarmingly, large amounts of EDs are released into the environment each year, facilitating their widespread dispersion and biological impact (Guarnotta et al., 2022). In aquatic ecosystems, fish are especially vulnerable to lipid-accumulating chemicals, with higher EDs levels observed in predator species due to food-chain bioaccumulation (Windsor et al., 2019; Zhang et al., 2022).

More than 100 natural and synthetic EDs have been described as thyroid disruptors, affecting TH synthesis, secretion, and peripheral action (Rodrigues et al., 2024; Dsouki et al., 2024). Among EDs, bisphenols, triclosan, phthalates, flame retardants, and parabens stand out, with effects well documented in mammals and increasingly investigated in zebrafish, which has emerged as a valuable model for elucidating the molecular mechanisms by which these contaminants impair HPT axis function. Therefore, Figure 2 not only illustrates the HPT axis, TH synthesis, and their physiological roles in zebrafish, but also highlights the key parameters reported to be disrupted by EDs, which are discussed in detail in the following sections.

#### 1.2.1 Bisphenols

Studies indicated that bisphenol A (BPA) exposure reduces TH levels in zebrafish, leading to morphological and functional impairments in TH-regulated organs such as the retina, heart, and central nervous system at doses ranging from 1 µg/L to 4 mg/L (Qin et al., 2023; Volz et al., 2024). Furthermore, *in vitro* screening assays show that BPA can both activate TH-receptors and disrupt TH binding to TTR, indicating stage-dependent agonistic and antagonistic effects on TH signaling in zebrafish (Li et al., 2023).

Interestingly, it has been demonstrated that BPA substitutes may also disrupt TH synthesis/secretion and action. Therefore, larval exposure to BPF, BPS, and BPZ up to 120 hpf altered both TH levels and gene expression profiles, inducing effects comparable to those observed with BPA exposure (Lee et al., 2019). Consistently, exposure of zebrafish embryos to low doses of fluorene-9-bisphenol (300–4,500 nM) up to 144 hpf induced a non-monotonic HPT axis response and disrupted myelination by altering TH levels and TH-regulated gene expression (Jin et al., 2021). Interestingly, the exposure to BPAP (18.2–105.9 μg/L) for 120 h reduced T_4_ levels only at the highest non-lethal concentration, with no effects on T3 or TH-related pathways, indicating a low potential to disrupt thyroid function (Lee et al., 2020).

In adult zebrafish, exposure to BPF (10–100 µg/L) and BPS (100 µg/L) increased TTR expression in multiple tissues, such as plasma, liver, and brain. In addition, both bisphenols elevated TH levels and damaged the structure of thyroid follicles, particularly in female exposed animals (Zheng et al., 2024).

In summary, these findings indicate that BPA analogues disrupt thyroid function in zebrafish with effects comparable to those of BPA. However, few studies have evaluated their impact on thyroid development, gene expression, or mixture effects that better reflect human exposure scenarios.

#### 1.2.2 Triclosan

The exposure of zebrafish embryos to the antibacterial agent triclosan (TCS) (30–900 ng/mL) up to 120 hpf has been linked to reduced hatching rates and histopathological alterations in thyroid follicles, impairing TH synthesis and secretion (Tang et al., 2022). More recently, combined exposure to TCS (20–80 µg/L) and the UV filter benzophenone-2 (2–7 mg/L), from 1 to 5 days post-fertilization, induced thyroid follicular hyperplasia and altered retinal structure in transgenic zebrafish embryos, demonstrating combined effects of EDs on thyroid organogenesis and TH-responsive organs during early development (Kraft et al., 2022).

In addition, environmentally relevant TCS doses (10.9–149.2 µg/L) increased expression of the *dio1* gene, suggesting a direct impact of this contaminant on TH metabolism and action in peripheral tissues (Horie, 2023). Interestingly, Wang et al. (2023) showed that TCS exposure during larval stages altered neurodevelopmental genes via epigenetic and neurotoxic mechanisms, but did not assess TH levels, which are critical for central nervous system development and function. Additionally, TCS exposure (0.5–2.5 µM) rapidly inhibited TH-related gene expression in zebrafish liver cells (Zhou et al., 2017).

Consistent with multigenerational effects reported in mammals (Henrique et al., 2025), exposure to TCS at environmentally relevant concentrations (1–10 μg/L) disrupted TH homeostasis in zebrafish by impairing thyroid axis regulation and thyroglobulin interactions, with effects persisting across generations (Ding et al., 2025).

Together, these findings demonstrate that TCS, even at environmentally relevant concentrations, acts as a potent thyroid disruptor in zebrafish, with effects extending beyond directly exposed individuals to subsequent generations. Considering the increased environmental release of TCS following the COVID-19 pandemic, these results raise serious concerns about its long-term endocrine and ecological impacts on aquatic organisms.

#### 1.2.3 Phthalates

Zebrafish embryos exposed to low doses of di-(2-ethylhexyl) phthalate (DEHP) or its metabolite mono-(2-ethylhexyl) phthalate (MEHP) up to 168 hpf presented reduced T_4_ levels and high T_3_ levels, accompanied by alterations in the expression of key HPT axis genes, such as those involved in the synthesis, peripheral metabolism, and transport of TH (Zhai et al., 2014; Jia et al., 2016). Consistently, both conventional phthalate esters [di-*n*-butyl phthalate (DBP) and diisobutyl phthalate (DiBP)] and their alternatives [diisononyl phthalate (DiNP) and diisononyl hexahydrophthalate (DINCH)] disrupted TH homeostasis in zebrafish embryos, mainly by inhibiting deiodinase activity and reducing T_3_ levels during early development (Tan et al., 2023). In agreement, alternative phthalates such as DiNP and di-(2-ethylhexyl) terephthalate (DEHTP) disrupted HPT axis function and behavior in zebrafish, with larval DiNP exposure (0.3–6.0 mg/L) increasing TH levels and altering neurodevelopmental genes, and DEHTP (10–1,000 µg/L) affecting sex hormone and TH levels across life stages (Ihn et al., 2024a; Ihn et al., 2024b).

Furthermore, a study comparing the individual and combined toxicity of DBP (0.05–5 mg/L) and its widely used substitute DiBP (0.1–10 mg/L) in zebrafish embryos demonstrated that both compounds disrupt the HPT axis by increasing TH levels and upregulating TH-related genes. Notably, combined exposure elicited synergistic toxic effects, and molecular docking analyses further suggested direct interactions of both phthalates with TH receptors, reinforcing their potential as thyroid disruptors in aquatic organisms (Tao et al., 2024).

Interestingly, a recent study confirmed that early-life exposure to DBP (0.001–1 mg/L) up to 144 hpf induced developmental toxicity and disrupted the HPT axis, leading to impaired growth, altered T_3_ and T_4_ levels, increased inflammation and oxidative stress, and dysregulation of key HPT axis-related genes. Notably, astaxanthin supplementation effectively mitigated these adverse effects by restoring TH homeostasis, normalizing HPT axis gene expression, and activating antioxidant and anti-inflammatory pathways through multi-target molecular interactions (Yu et al., 2026). Collectively, these findings suggest that the deleterious effects triggered by phthalates and other EDs may be effectively mitigated through antioxidant-based interventions.

In summary, while phthalates and their substitutes are known to disrupt the HPT axis in zebrafish, the molecular mechanisms underlying altered TH production and peripheral action remain poorly defined. This gap highlights the need to extend aquatic toxicology studies beyond DEHP to its emerging substitutes, as phthalates appear to target multiple regulatory steps of TH synthesis, metabolism, and transport, with systemic consequences that still require clarification.

#### 1.2.4 Flame retardants

Flame retardants are widely detected in human tissues and have been correlated with systemic endocrine alterations, including disruption of the HPT axis (Gao et al., 2024). Consistently, exposure to environmentally relevant levels of the polybrominated diphenyl ethers (PBDEs) BDE-47, BDE-99, and BDE-209 up to 96 hpf disrupted TH-related gene expression and induced lethal, developmental, and morphological abnormalities in zebrafish embryos (Zezza et al., 2019).

It is worth noting that available evidence indicates PBDE metabolites can be as toxic as, or even more toxic than, their parent compounds. Accordingly, studies have shown that exposure of zebrafish embryos to the metabolite 6-OH-BDE-47 (1–100 nM), which also shares structural similarity with TH, induces apoptosis in retinal cells and reduces ocular pigmentation. These effects were reversed by TH receptor beta (*thrb*) overexpression, indicating a TH receptor-mediated mechanism induced by 6-OH-BDE-47 exposure (Dong et al., 2014). Similarly, a study demonstrated that early-life exposure of zebrafish embryos to 6-OH-BDE-47 (50–100 nM) induced neurodevelopmental toxicity, characterized by increased coiling behavior and elevated neuronal apoptosis. Notably, these effects were partially rescued by *thrb* overexpression, reinforcing that TH signaling is a key pathway of 6-OH-BDE-47-induced neurotoxicity (Wang et al., 2018).

Macaulay et al. (2015) further confirmed that this metabolite (1–250 nM) causes severe developmental delays, characterized by a reduced head-trunk angle, increased optic vesicle length, and decreased eye pigmentation, parameters that are regulated by nuclear TH receptors. Similarly, PBDE metabolites exposure, either individually or in mixtures, has been shown to upregulate genes involved in skeletal development and TH regulation, adversely affecting the zebrafish maturation and metamorphosis (Macaulay et al., 2016). Additionally, exposure to 6-OH-BDE-47 (1 nM) and 6,6′-diOH-BDE-47 (20 nM) disrupted TH regulation in zebrafish larvae, as evidenced by increased TH levels and upregulation of thyroid-specific genes, suggesting the activation of the HPT axis. Therefore, despite lower toxicity, 6,6′-diOH-BDE-47 still posed endocrine-disrupting risks to the HPT axis at environmentally relevant concentrations (Zhang et al., 2021).

Recent evidence indicates that organophosphate esters (OPEs) also disrupt the HPT axis in zebrafish through multiple mechanisms. In this context, integrated *in vivo, in vitro,* and *in silico* studies show that OPEs interfere with TH transport, synthesis, and receptor-mediated signaling, leading to impaired TH availability and dysregulation of thyroid-related gene expression (Yan et al., 2022). In fact, exposure to tris (2-butoxyethyl) phosphate (TBOEP) at 100 and 500 µg/L markedly suppressed the expression of multiple genes across the HPT axis, including regulators of TH synthesis, transport, metabolism, and signaling (Dong et al., 2025).

Importantly, recent studies indicated that flame retardants elicit multi- and transgenerational effects, extending their impact beyond directly exposed individuals and affecting subsequent generations. Evidence from adult zebrafish demonstrated that chronic exposure to BDE-209 (3–300 μg/L), but not lower-brominated congeners, resulted in significant bioaccumulation, maternal transfer to F1 embryos, and teratogenic outcomes in the offspring. These effects were accompanied by marked reductions in T_4_ levels at 120 hpf and dysregulation of HPT axis-related genes, further supporting the capacity of highly brominated PBDEs to disrupt TH-dependent developmental processes (Han et al., 2017). Consistently, lifelong exposure to the brominated flame retardant DBDPE (0.1–10 nM) induced epigenetic HPT axis alterations that were transmitted across generations in zebrafish, indicating transgenerational endocrine disruption (Sun et al., 2024). Finally, TBOEP accumulated in F0 adults and was maternally transferred to F1 embryos, inducing dose-dependent developmental toxicity, including increased mortality, deformities, and impaired cardiac and swimming functions. These effects were accompanied by reduced egg protein content and downregulation of GH/IGF and HPT axis-related genes (Dong et al., 2025).

#### 1.2.5 Parabens

Butylparaben (1–2.5 mg/L) and ethylparaben (5–30 mg/L) exposure during the embryonic period caused harmful effects in zebrafish, including increased mortality, reduced hatching rate, decreased body length, lower heart rate, and a higher incidence of malformations (Merola et al., 2020). In agreement, it has been demonstrated that parabens (2–200 µM) exposure up to 120 hpf reduced TH levels by disrupting the expression of HPT axis-related genes. Moreover, molecular docking analyses showed that methyl-, ethyl-, propyl-, and butylparabens exhibited binding affinity for TH receptors, acting as partial agonists with a potency that increased in parallel with alkyl chain length (Liang et al., 2022). Dasmahapatra et al. (2024) reinforced the structure-activity relationship, demonstrating that parabens with longer alkyl chains exhibit greater toxicity in fish, leading to lethal effects, multisystemic EATS (estrogenic, androgenic, thyroid, and steroidogenic) disruption, intergenerational toxicity, and impaired embryonic development.

Interestingly, integrated transcriptomic and metabolomic analyses in zebrafish revealed that paraben exposure disrupts tyrosine metabolism, a key pathway for TH synthesis, thereby linking thyroid dysregulation to broader systemic metabolic disturbances (Wei et al., 2024).

Together, these findings establish parabens as EDs of the HPT axis in zebrafish, with effects comparable to those in rodents. However, major gaps remain regarding long-term, low-dose, and mixture effects, and zebrafish offers a critical model to clarify the mechanisms and risks of paraben exposure across developmental and generational windows.

#### 1.2.6 Other EDs and mixtures

Beyond data on classical thyroid-disrupting EDs, recent zebrafish studies have identified emerging contaminants and chemical mixtures that interfere with HPT axis function.

Therefore, exposure of zebrafish embryos to liquid crystal monomers used in LCD screen manufacturing has been shown to damage retinal structures and disrupt TH levels by altering the expression of key HPT axis proteins (He et al., 2024). In addition, exposure of zebrafish embryos and larvae up to 120 hpf to organic UV filters widely used in cosmetics, including avobenzone (1–30 µM), benzophenone-3 (0.04–1.40 µM), octocrylene (1–30 µM), and octyl methoxycinnamate (1–30 µM), has been associated with thyroid toxicity, and neurobehavioral and renal alterations, reinforcing that thyroid dysfunction may underlie multiple systemic adverse effects in the organism (Ka and Ji, 2022; Reum Kwon et al., 2024).

It is worth noting that humans and other animals are exposed to mixtures of EDs rather than to single compounds (Pearce, 2024).

In this context, 10-day-old zebrafish larvae exposed to river sediments naturally contaminated with TCS, galaxolide, polychlorinated biphenyls, BPA, and other substances exhibited multiple alterations in the expression of genes involved in thyroid gland development regulation, TH synthesis, metabolism, transport, and action in target cells (Viganò et al., 2020). Additionally, recent studies show that exposure of zebrafish embryos to mixtures of polystyrene microplastics with BPS and MEHP, even at individually non-toxic levels, induced synergistic embryonic damage, associated with oxidative stress, disruption of TH synthesis-related genes, and activation of stress- and damage-related markers (Kim et al., 2024).

Thus, although defining relevant doses and exposure windows for reliable translation to human health remains challenging, there is a clear need for studies on ED mixtures, as they better reflect real-world exposures and can elicit adverse effects at lower doses than individual compounds.

### 1.3 Challenges of the zebrafish model

Despite its many advantages, the zebrafish model presents important technical and biological limitations that must be considered during experimental design and data interpretation. High intragroup variability is frequently reported, even under controlled conditions, and is influenced by individual physiology, genetic background, strain-specific behaviors, and epigenetic factors such as parental stress history (Faught et al., 2016; Abozaid et al., 2022).

In addition, the small body size of zebrafish, particularly at early developmental stages, limits tissue availability for molecular and histological analyses, a challenge compounded by technical difficulties in sectioning embryos and larvae and by the still-limited repertoire of validated antibodies for zebrafish proteins (Pérez Díaz et al., 2025).

Further constraints include the ectothermic nature of zebrafish and their optimal development at 28 °C, which may affect thermosensitive phenotypes and reduce direct translational comparability with mammals (Miller et al., 2025). Variability among strains, lack of standardization of biological material, use of commercially sourced fish with genetic divergence from reference genomes, and potential contamination in metagenomic approaches can also compromise reproducibility (de Abreu et al., 2024; Gerlai, 2019).

Finally, breeding efficiency is sensitive to environmental and husbandry conditions, and multicenter studies have shown that subtle differences in water quality, lighting, noise, handling, and housing significantly impact behavioral outcomes (Hillman et al., 2024). Given that behavioral endpoints are commonly used to assess ED-induced disruption of the HPT axis, rigorous control of these methodological factors is essential to ensure robust, reliable, and reproducible zebrafish-based studies.

## 2 Conclusion

Zebrafish has become a well-established model for investigating thyroid embryonic development and function, as well as the actions of TH on multiple physiological systems. The high similarity of the zebrafish endocrine system to that of higher vertebrates, combined with its genetic manipulability and embryonic transparency, has enabled significant advances in understanding the deleterious effects triggered by EDs exposure, particularly within the HPT axis. Recent studies have expanded the use of this model to investigate multi- and transgenerational effects of EDs on TH-mediated regulation of peripheral systems. Consequently, the zebrafish model has emerged as a valuable tool for elucidating the molecular mechanisms underlying environmentally induced thyroid dysfunctions; however, challenges remain in accurately modeling human-relevant exposure doses and critical windows of exposure.
